# A Culturally Humble Approach to Designing a Sports-Based Youth Development Program With African-Australian Community

**DOI:** 10.1177/10497323241231856

**Published:** 2024-03-14

**Authors:** Rachel Goff, Patrick O’Keeffe, Abraham Kuol, Rob Cunningham, Ronnie Egan, Bawa Kuyini, Robyn Martin

**Affiliations:** 1 5376 RMIT University, Melbourne, VIC, Australia; 2 Afri-Aus Care, Melbourne, VIC, Australia

**Keywords:** community-based co-design, UBUNTU, research methods, cultural humility, culturally diverse communities, grassroots community design, decolonization, social work

## Abstract

This article draws on the concept of cultural humility, to describe and analyze a decolonizing approach to co-designing a primary prevention basketball program for young African-Australian people in Melbourne, Australia. We explore the potential for genuine collaboration and power-sharing with a culturally diverse community through collaboratively developing the co-design process and resultant program design. This article highlights the central role of UBUNTU in the co-design process, prioritizing African ways of knowing, being, and doing within a Westernized social work and design context. Through reporting on the stages of program design, we offer an example of how Indigenous knowledges and philosophies such as UBUNTU might be incorporated into co-design through cultural humility. We suggest this allows for a transformation of design tools and processes in ways that undermine oppressive and marginalizing power imbalances in design and social work.

## Introduction

Engaging with culturally diverse communities has become a central theme in design research and in the development of programs and services intending to address the often nuanced and distinct needs encountered by culturally diverse communities (Kapuire et al., 2018; Winschiers-Theophilus et al., 2012). It is argued that Western researchers should aim to decolonize their design practices by attending to settler-colonial power relations, partnership, and collaboration with the migrant and resettled communities they are designing with (Smith et al., 2020; Winschiers-Theophilus et al., 2012). Such partnership and collaboration attend to the broader social, cultural, and philosophical context experienced by the community, leading to holistic, and culturally informed interventions and outcomes (Frounfelker et al., 2020). Community-based co-design (CBCD) (Till et al., 2022) methodology can support researchers to actively partner with communities through the application of co-design principles, processes, and methods in a community setting while emphasizing community needs, practices, and values within the research. Numerous case studies describe design projects with culturally diverse communities (Carr et al., 2021; Kambunga et al., 2020), yet there are limited scholarly examples describing how researchers adjust their practices in cross-cultural research to de-center the methodological decision-making power afforded to them from the academic context.

Efforts to decolonize design research first requires us to acknowledge the institutional and colonial power we occupy as researchers, and interrogate the theoretical inadequacies of Western design research, a practice Moosavi (2023) describes as “decolonial reflexivity.” We are a team of researchers who inhabit diverse cultural identities, signifying various proximities to the dominant, Western-centric worldview. We also identify as critical social work and community researchers who have varied practice and research experiences in culturally diverse settings, as well as in other contexts with structurally vulnerable communities. Because of our research and professional experiences, we recognize that the social work discipline has a legacy of and continues to participate in systems of oppression, such as the colonial project of Indigenous child removal (Joubert & Webber, 2020). While we are practiced in participatory methodologies and designing with communities, we also possess decision-making power and the ability to privilege certain knowledge to reach research outcomes because of these academic roles (Joubert & Webber, 2020). These factors compel us to critique our own practices and be accountable to the communities with whom we research, particularly in how we aspire to respect, partner, and intentionally attend to and disperse our power.

This paper describes our practice of cultural humility (Tascón & Gatwiri, 2020), defined as a “process of openness, self-awareness, being egoless, and incorporating self-reflection and critique after willingly interacting with diverse individuals” (Foronda et al., 2016, p. 213). By engaging with cultural humility in CBCD research, researchers are prompted to attend to power differences between themselves and other stakeholders, enable an environment where all stakeholders can equitably collaborate, work in ways that are appropriate to the community, and generate a greater sense of ownership within the community in more aspects of the design process (Till et al., 2022).

This paper begins with a description of cultural humility, including its practical and methodological implications when practiced alongside the African philosophy, UBUNTU, an African philosophy of life, humanity, individuality, and community (Kapuire et al., 2018). We then extend our decolonial reflexivity (Moosavi, 2023) to a critique of Western design practices, which appear to universally create authentic collaboration, partnership, and power-sharing, describing how a culturally humble CBCD methodology challenges settler-colonial design practices by attending to these principles with greater nuance. Next, we describe the CBCD project that intends to prevent youth crime by enhancing UBUNTU in an African-Australian community in Melbourne, and we offer examples that demonstrate our aim of cultural humility. We conclude by reflecting upon our learnings from the design process and aim to demonstrate how cultural humility helps us to work with issues of power, collaboration, reciprocity, responsiveness, and curiosity to re-position our role as partners rather than experts.

## A Brief Introduction to Cultural Humility: The Example of UBUNTU

Cultural humility is a concept, theory, and practice emerging from cultural care and competency theories in the healthcare profession (Foronda, 2020). Cultural humility requires lifelong reflection and action whereby researchers continuously self-evaluate and self-critique to redress hegemonic power imbalances resulting from colonization or Westernized notions of cultural difference (Foronda, 2020; Tervalon & Murray-García, 1998). Unlike cultural competence, which assumes respect and esteem for cultural diversity is learned and achieved in measurable steps for cultural proficiency (Foronda, 2020; Tascón & Gatwiri, 2020), cultural humility is a dynamic exchange between researchers and community members resulting in mutual empowerment, respect, and partnership (Foronda et al., 2016). According to Tervalon and Murray-García (1998, p. 118), cultural humility is embodied by admitting “we do not know what we truly do not know,” rather than claiming expertise about a defined population’s culture or preferred ways of interacting or relating.

A key principle of cultural humility is acknowledging and redressing power imbalances between researchers and the community involved in the research. A mindset of *not knowing* draws attention to racialized power relations and challenges traditional researcher roles by enabling mutual curiosity and non-judgment and in doing so abrogates a researcher from the expectation to demonstrate hierarchical cultural knowledge or proficiency *about* the community they are engaging in the research process. Instead, cultural humility emphasizes an open partnership approach with the community through deep listening and responsive action. These actions also act to deemphasize researcher’s personal experiences or subjective interpretation of culture (Tascón & Gatwiri, 2020). Therefore, culturally humble practice has the potential to create the necessary conditions for collaboration inherent to CBCD research.

We adopted a culturally humble approach to CBCD in our learning about the UBUNTU philosophy that is the guiding worldview of the African-Australian community members involved in the research. UBUNTU is an African epistemology grounded in the collective values and practices of African people. While the practice of UBUNTU varies across cultural and ethnic groups, UBUNTU recognizes an individual as a part of a larger and more significant relational, communal, societal, environmental world (Mugambate & Chereni, 2020). UBUNTU promotes inclusion, participation, relationship strengthening, reciprocity, and belonging for collective well-being and success (Winschiers-Theophilus et al., 2012). UBUNTU-focused design research (Kapuire et al., 2018; Smith et al., 2021) aims to design with a community in ways that are epistemologically, theoretically, and relationally grounded in these values. This article illustrates our attempts to prioritize power-sharing, partnership, reciprocity, collaboration, and responsiveness to develop, with the community, a culturally appropriate research process and design a sports-focused youth crime prevention program that is grounded in community understanding and practices of UBUNTU.

## Tensions in Co-Design: Not a Neutral Space

Situated within discourses of innovation and creativity, co-design is espoused as a universally accessible and replicable methodology to create ‘positive impact’ with and in community settings (Akama et al., 2019). In both research and service design, designers commonly follow an established convergent and divergent process and creative methods to engage with people, understand their problems, and establish solutions that meet their needs. However, such objective and replicable methods were first established in a Western setting and often do not explicitly address or transform power relations that may emerge from Westernized, colonial worldviews or during the process (Hernandez Ibinarriaga & Martin, 2021). Agid and Akama (2018) criticize ‘off-the-shelf’ methodologies or ‘toolkits’ because a universal approach may not consider the values, knowledge, and epistemologies informing various non-Western worldviews of the co-design team (researchers, community, or others); nor do they explicitly attend to reflexivity, curiosity, or concerns of relationality and power relations that exist from a culturally humble standpoint.

It is essential to critically reflect on the apparent neutrality of design, particularly when utilized in culturally diverse contexts. Scholars suggest that design may reinforce individualization and deficit-based approaches, which can further marginalize Indigenous epistemologies (Abur & Mugumbate, 2022), such as UBUNTU. Further, settler-colonial hegemony may be reinforced when designing without adequate knowledge of local knowledge systems or cultural realities (Smith et al., 2020; Winschiers-Theophilus et al., 2012). Given the burgeoning popularity of co-design in Western contexts, it is unethical to believe that ‘universal’ practices will naturally translate across all communities; such an assumption does little to challenge the settler-colonial conceptualization of Westernized thought and knowledge as preeminent. For research aiming to challenge systems that reproduce settler-colonial power relations that marginalize non-Western or Indigenous peoples and knowledges, it is essential to re-think and enact design practices that intentionally challenge aspects of coloniality such as white privilege and subconscious bias (Kambunga et al., 2020; Kapuire et al., 2018).

We join a growing body of researchers aiming to decolonize design as it grows in popularity in culturally diverse settings. Studies suggest that ‘designing with’ communities may extend to the inclusion of mutual learning and respect of cultural protocols that promote dialogue and inclusive decision-making in the initial stages of community engagement (Kapuire et al., 2018; Smith et al., 2020; Winschiers-Theophilus et al., 2012). These protocols may relate to community ownership over design activities or tools, such as how a community is entered and interacted with, or understanding cultural communication styles which dictate how a design tool may be used—or not (Kapuire et al., 2018). Specific to designing with African-Australian communities, designing with, and not for people, is considered an inclusive way of restoring power to people and communities who have experienced discrimination by Westernized institutions (Shepherd et al., 2022; Smith et al., 2021) both during migration and resettlement.

Scholars also describe how the discrimination and disenfranchisement that African-Australians have experienced extends to the development and delivery of services and programs (Shepherd et al., 2022). Mainstream services are often culturally unresponsive and inflexible (Gibbs & Block, 2017; Shepherd et al., 2022). In a sporting context, young people engaging with mainstream sports clubs and organizations experience pedagogies of exclusion, through racialized discourses not applied to white, middle-class, and able-bodied young people (Gibbs & Block, 2017). Shepherd et al. (2022) suggest that designing culturally informed, early intervention programs for young African-Australian people and their families is essential to providing holistic support that can strengthen community connection and disrupt stigmatizing and exclusionary narratives inherent to settler-colonialism and Westernized institutions.

Infusing cultural humility with CBCD is significant for two key reasons. First, when adopting a stance of curiosity and critical reflection, design researchers can recognize the limitations of their own cultural knowledge and identify personal biases and positionality in relation to co-researchers (Tascón & Gatwiri, 2020). Second, by acknowledging and emphasizing the cultural expertise of community co-researchers, academic design researchers can consciously redistribute their inherited power within the design process to involve and partner with community members in all research development processes (Agid & Akama, 2018; Kapuire et al., 2018), including the development of engagement protocols and co-design tools which can then more powerfully align to a community’s ways of knowing, being, and doing. As such, culturally humble practice may facilitate decolonization in CBCD because it challenges researchers to de-center their own knowledge, privilege, and mastery of the methodology to focus on the quality of relationships developed with community.

With these ideas in mind, this article now moves to illustrate our embodiment of cultural humility in the development of an UBUNTU sports-based youth-development basketball program with a Melbourne-based African-Australian community. While the UBUNTU philosophy is recognizable in co-design studies in African contexts, there are no known studies that reflect upon culturally humble CBCD with resettled communities in Australia, nor in sports-based program design. This paper seeks to contribute to filling this gap.

## Study Context

### The Project

This article reflects on the authors’ practice of cultural humility in the design of the expanded Black Rhinos Basketball Program for primary and early secondary school–aged children and their families. The Black Rhinos Basketball Program is a sports-based youth-development program. These programs have demonstrated capacity to facilitate social inclusion and belonging and increase psychosocial well-being for young people from refugee, migrant, and diverse backgrounds (Bunde-Birouste et al., 2012). The Black Rhinos primarily focuses on crime prevention and was developed by grassroots resettlement support service Afri-Aus Care, for young African-Australian people (aged 15–25) in Melbourne, Australia. This program is underpinned by the African philosophy of UBUNTU, which intends to connect young African-Australian people with community, culture, and family, in ways that support health and well-being, reduce isolation, and provide holistic support and care. The project is funded through the Victorian Health Promotion Foundation (VicHealth) and sits within health promotion funding as research highlights that young people from refugee backgrounds are at greater risk of social isolation, poor health outcomes, language and communication barriers, disengaging from school, and experiencing unemployment, mental health concerns, poor living conditions, substance misuse, and criminal justice involvement (Bunde-Birouste et al., 2012; Cunningham et al., 2020). The project drew upon an established relationship between Afri-Aus Care and the university’s Social Work Field Education Program.

## Method

The examples illustrating our practices of cultural humility result from our involvement in a community-based co-design (CBCD) project, which draws upon participatory design and action research to identify local strengths, needs, and ‘fit-for-purpose’ solutions relevant to a design project (Winschiers-Theophilus et al., 2012)—in this case, expanding the Black Rhinos Basketball Program for a younger age group. CBCD is informed by the specific protocols present within a community and aims to acknowledge power differences between the community and other stakeholders and in doing so attempts to establish the conditions for more equitable community ownership, collaboration, and leadership within the research (Till et al., 2022). Ethical approval for this study was granted by the RMIT University Ethics Committee, and all university researchers participate in ongoing RMIT University ethics compliance training modules.

### University Research Team/Authors

The research team consisted of six RMIT University researchers who were practiced in community-based and participatory research and research partnerships, co-design, and sports-based youth-development programs. The team also included regular contributions from the Afri-Aus Care project manager who has experiential and professional knowledge of the UBUNTU framework and its application in community programs, who is identified in the article as an agency ‘co-researcher’. Two research team members are of African background, and their contributions to the team included cultural and organizational advice as well as direction on integrating UBUNTU into the initial research design development. The university research team are the authors of this article.

### Co-Researchers

Recruitment of community participants, whom we refer to in this article as ‘co-researchers’ to reflect the intention to create respectful partnership with community, was managed by the agency program manager. Co-researchers included men and women of different ages and standings in the community, who are either community elders and leaders or young people with experiences of the Black Rhinos program who are seen as emerging leaders (Table 1). Of the 17 co-researchers, 14 are from refugee backgrounds and arrived in Australia through Humanitarian visa programs and the remaining three arrived as skilled migrants. All co-researchers have lived experiences of acculturation challenges related to accessing the labor market and experiences of racism and discrimination. Following the provision of their details by the project manager, researchers contacted the potential co-researchers describing the activities in each phase of the process, and both written and verbal consent was provided prior to the research commencing. Co-researchers were reimbursed for each phase they engaged in with a $50 fuel voucher, which Afri-Aus Care identified as the best form of recognition of participants’ contributions. All co-researchers were de-identified, and other identifying information was removed. As research participants, the co-researchers were not required to complete RMIT University ethics training; however, a shared understanding of the ethical requirements of the research was developed through formal (e.g., consent gathering) and methodological processes (e.g., partnering to shape research process, described below).

**Table 1.** Co-Development of Design Phases.

Phases	Purpose	Who Was Involved
Phase 1: Discover focus groups	• Establishment of relationship with co-researchers • University researchers and co-researchers develop shared understanding of UBUNTU philosophy and cultural protocols • Co-researchers reviewed, added, or refined proposed interview activities and design tools	• Group 1: 10 community leaders/elders or parents of children who were involved in Afri-Aus Care activities • Group 2: 7 young people, aged 18–25, with experiences of the existing Black Rhinos program or other sports-based programs
Phase 2: Analysis	• Focus groups transcribed and thematically analyzed by the research team • Activities and tools reviewed and modified in line with community feedback	• University researchers
Phase 3: Define exploratory interviews	• Explored co-researchers’ experiences of health and well-being, community, and program impact measures • Design tools utilized if co-researchers agreed • Prompt questions guided discussion	• 14 co-researchers (7 individual interviews, 2 interviews with 2 co-researchers, and 1 interview with 3 co-researchers)
Phase 4: Analysis	• Focus groups transcribed and thematically analyzed by the research team • Interview transcript reviewed by researchers	• University researchers and co-researchers
Phase 5: Design workshop	• Findings discussed with co-researchers • Researchers facilitated design charrette to confirm and explore the community’s ‘big ideas’ for program implementation	• 15 young people and community elders/leaders with previous involvement in the program

### Design Phases

The first three stages of the UK Design Council’s (2023) Double Diamond were adapted for the initial research design. These stages—discover, define, and design—enabled researchers, Afri-Aus Care, and co-researchers to jointly articulate issues of importance, define how the program could strengthen their community through UBUNTU, and develop inspiration for the expanded program (UK Design Council, 2023). The use of the Double Diamond also provided a ‘roadmap’ for the researchers in the initial planning stages, acting as a framework to embed the collaborative and participatory principles of the CBCD methodology (Till et al., 2022) into activities, including the choice of research methods.

With Afri-Aus Care staff and representatives from the Black Rhinos program, we developed a co-design process for designing the expanded Black Rhinos program. Aligned with the community-led principle of CBCD (Till et al., 2022), an initial focus group allowed the co-researchers to share their ideas for how they would like to participate in other research activities. The co-researchers communicated their preference for storytelling to other qualitative methods, and as such, the focus group determined that interviews and a concluding focus group would facilitate narrative and open-ended techniques to define program priorities and develop key elements. Such qualitative methods also facilitate the use of design tools. Design tools are common in CBCD to elicit research insights from the perspective of community members, although they are generally designed and used uncritically and without input from those who ultimately use them (Agid & Akama, 2018). Table 1 details the co-development and use of these tools, illustrated in the examples below.

### Data Collection and Analysis

Focus groups and interviews were audio-recorded and transcribed by the researchers. Data was used to inform each subsequent stage of the co-design process. Transcripts were thematically analyzed by the researchers using sensitizing concepts relevant to the purpose of the research (UBUNTU, community, health and well-being, and program characteristics), who then met with Afri-Aus Care staff to plan the subsequent stage of program development. The research team reported the analysis back to community members to clarify key themes and finalize program aims and objectives. The research project did not have sufficient resources to support co-analysis of data with the community. In addition, ethics requirements meant that we were unable to share transcripts beyond the research team. In the following section, we provide examples of the process of developing co-design tools with community, in ways that aimed to be culturally humble. We also illustrate how this approach utilized UBUNTU as a primary lens through which to understand community, health, and well-being, which could then be applied by community and researchers to develop a culturally responsive program.

## Cultural Humility and Design in Action: Principles and Examples in the Black Rhinos Project

In this section, we connect our intentions as researchers with examples of how we enacted this in the research design and implementation. We include analysis of preliminary discussions between the research team where we sought to establish alignment between principles of the research and design process and our understanding of cultural humility, analysis of aspects of the focus groups, and interviews which reflect the enactment of this approach to cultural humility and how it has shaped the design process.

### Relationships

#### Sharing Power

In relation to Tascón and Gatwiri’s (2020) discussion of the centrality of committed relationships founded on care, respect, and mutual learning, our approach to cultural humility centers on the development of relationships where power-sharing, trust, care, and reciprocity are priorities. Through our preliminary discussions as a research team, we considered how power could be shared through the co-design process. First, we reflected on the Westernized ideologies and knowledge inherent within co-design, drawing on Smith et al. (2020), Kapuire et al. (2018), and Winschiers-Theophilus et al. (2012). We aimed to decolonize by recognizing the limits of our own knowledge of UBUNTU and African cultures and being curious and respectful. This assisted in redistributing and conceptualizing expertise. A significant element of our approach was to prioritize UBUNTU as a guiding philosophy for the project. Through the co-design focus groups, we asked community leaders and young people to describe how they understood UBUNTU and how they saw UBUNTU enacted within their communities. To stimulate the conversation, we first shared what we understood about UBUNTU but acknowledged that we did not yet know what it meant to co-researchers. We asked the co-researchers to spend 5 min thinking about how they characterized UBUNTU and to then record their thoughts and share responses. As part of this process, we encouraged storytelling as a way for co-researchers to convey what UBUNTU meant to them. This is illustrated by one co-researcher in their description of UBUNTU:

It is showing love and unity, that’s the way of UBUNTU ... When we start in 2019, it was terrible. We didn’t have unity or a big table like this ... We didn't have food to eat. Sometimes we are missing things ... Sometimes [community leaders at Afri-Aus Care] would cook for us, potatoes with salt, and then we eat. That’s it. And we are happy ... Mama T, Mama S, they help in 2020 and then we had some food especially in the time of Coronavirus, 2021, it was very shock and hard times, but all the young kids, the Black Rhinos, they been going to house to house to [bring] the food. They been going to Pakenham, Narre Warren, Dandenong here, for all the elders. And UBUNTU would come from Dandenong up to that area ... And that’s how we’ve been working together as UBUNTU works.

Through creating opportunities for storytelling, and centering UBUNTU within the project, we shifted from individualized, Westernized practices, ideologies, and knowledge. Our intention was to work toward an approach to thinking and communicating that aligned with the complex and multiple conceptualizations and practices of UBUNTU, which is united by certain elements of the philosophy but differentiated by lived experiences and context.

#### Reciprocity

Connected to the intention to share power and establish trusting relationships, a focus on creating reciprocal relationships was another key element of our co-design approach. Reciprocity is frequently described as integral to UBUNTU, as a value and in practice (Mugambate & Chereni, 2020). We considered a reciprocal relationship essential in deconstructing traditional power relationships between the researcher and the researched, focusing on researching with community. In thinking about reciprocity, we also considered how, as researchers, we could share something of ourselves. As this exchange between a university researcher and Afri-Aus Care staff member illustrates:

University researcher: And it’s very clear to me that we don’t want to do a traditional research approach where we go in and we mine people for data, and then we step out. Which is not really our team’s approach anyway, but it is certainly a very dominant approach. And what I mean by reciprocity, is that we ask people to tell us about a topic, them, their life, whatever. But what is it that we tell them about ourselves?

Agency co-researcher: Definitely agree, that is a really good approach. If someone can feel and understand that you care about what you are doing, or they know your intentions, then it’s like, “OK, this person wants to support this.”

Consequently, we created an interview structure that required university researchers to share their own story and background with community co-researchers. Another university researcher described the approach researchers took when visiting Afri-Aus Care to conduct research work:

For me, one of the things I often find quite rewarding in community engagement is going in there and being just, not very guarded. When you meet out there, you are very open and relaxed and making the conversation very informal, even though you have parameters around when you are going to finish.

In this regard, it is not just the intention to share power but also embodying an openness through informal actions, communication, and body language, which is integral to showing that the researcher is willing to share something of themselves with community.

Further, commitment to community was identified as a key aspect of reciprocity. In the focus group with community elders, co-researchers were asked about how they understood a reciprocal relationship:

University researcher: How do you think that we can be working together in ways that are reciprocal? So, like, obviously you are taking time out of your day to speak with us, which we really appreciate. What are the kinds of things that we can be doing as well?

Co-researcher: Probably just having that dialogue. So not just like today, then gone. Come back and do the interviews, then gone.

This reflects concerns about an extractive or transactional approach to research and program development and illustrates how reciprocity is understood in terms of ongoing commitment, interest in the community, and openness to dialogue and sharing.

#### Responsiveness

As a research team, we reflected on the potential for Westernized methods of communication, including verbal and non-verbal communication, as potentially alienating for co-researchers. Drawing on Kapuire et al. (2018), we discussed asking co-researchers about key ‘do’s and don’ts’ we should be abiding by as university researchers.

University researcher: It’s the invitation to let us know that we probably haven’t got it right. I think this is really well intentioned and I get where it is coming from, but I also think it’s in the doing, that people will then say, “Actually, that’s not OK.”

Agency co-researcher: Yes, because the intentions of the project are really good … People feel the energy and the vibe. When they feel like they are being exploited, they understand that. They are going to be able to feel the energy that you are giving off, the vibe that you are giving off. That’s when they will be hesitant. That’s when they will be really reacting negatively to the questions that you have.

In many ways, this relates to trust. Asking about the ‘do’s and don’ts’ is important in demonstrating an intention to be reliable and trustworthy. Though, as the agency co-researcher states here, the enactment of trustworthiness and the demonstration of culturally respectful and culturally informed behavior were central to developing a trusting relationship with community members. Thus, the ‘asking’ is significant in demonstrating intentionality, but the ‘doing’ is what would create the ‘vibe’ described above.

At the start of the focus groups, we asked co-researchers to tell us what we needed to know before starting the co-design process, including how to communicate and letting us know of cultural norms and values we should abide by. As one community elder co-researcher stated in their focus group, eye contact, which is expected in Westernized approaches to communication, can be challenging and confronting:

Before in our culture, you can’t talk to elderly people when you look in their eyes like this. That’s not respect. You put your head down when you speak with the elderly people. But in Australia, it’s a different way. When you put your head down, they say you are a criminal person. That shocked us. And that’s why you see a lot of young kids now, they don’t respect us, because when at school [teachers] tell them “Why put your head down? You have to put your eyes up.” And then they look at elderly people in the eyes when they come home, and that’s a big challenge for us.

Further, another community elder co-researcher stated that as researchers we had a role to play in ensuring cultural practices were maintained:

Can I say for the process of this focus group like the one we are practicing at this center, it’s like restoring our culture. Aunty, Uncle, Sister, and something. So if you try to practice that, that’s encouraging us as well.

### Co-Researchers Shaping the Research and Design Process

**Figure 1.**
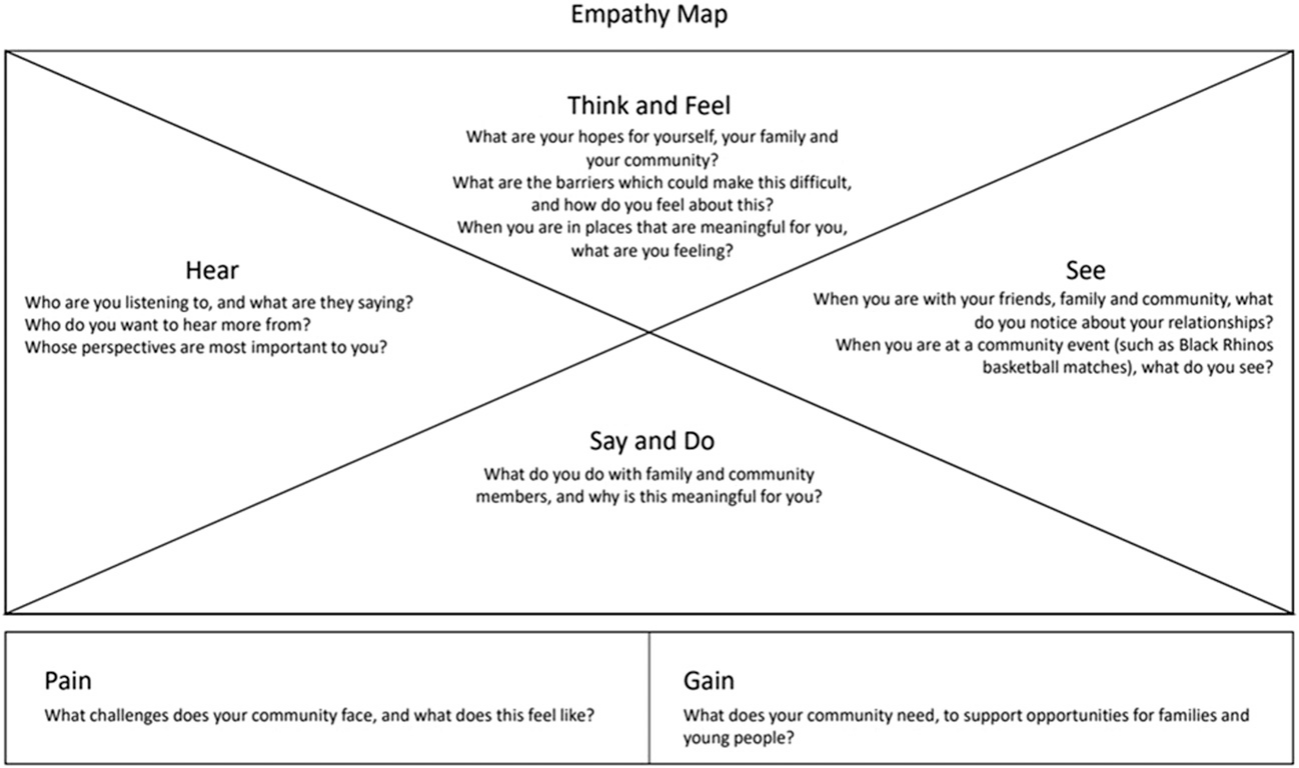
Empathy map, developed for focus group testing of co-design tools.

Reflecting on the critique of Westernized design tools as promoting a form of individualism, we developed an empathy map to help understand peoples’ experiences of community. Empathy maps are a visual mapping tool that synthesizes known information about an individual or group to assist in understanding another’s perspective (Cairns et al., 2021). The empathy map (Figure 1) is an attempt to integrate principles of UBUNTU into the development of a co-design tool. Through this tool, we sought to understand how community members think and feel and what they observe, experience, say, and do within their community, as a member of that community.

When the researchers showed the empathy map to the focus group featuring young people, co-researchers took some time to read and interpret what the tool was trying to achieve. Eventually, several co-researchers stated that the map included too much information. This led to a group reflection on the effectiveness of the tool:

Co-researcher: Even looking at it now, I am looking at it and like “What?”

Co-researcher: A conversation ... is better, because maybe as you go you can come up with more things to speak about. Whereas like this …

Co-researcher: Maybe simplify it ... So the focus is just on one thing at a time. Because with this map, as everyone is expressing, your mind might actually be all over the place. You’re like “Damn!”

As the extract shows, the young co-researchers clearly expressed their responses to the empathy map and, however, did not necessarily critique the questions raised within the map. Further, the young people provided constructive feedback that enabled a clearer, more culturally appropriate articulation of the ideas expressed within the empathy map. The co-researchers show a preference for a conversational, narrative-based approach, with questions as conversational prompts. Further, one young person suggested a photo elicitation approach to exploring themes of community:

What if I suggested that instead of there being like words, there would be like a picture that you could see. And then it helps them go “Wow, OK, how do I feel about this, what might I hear?”

The focus group discussed possible images that they connected with the community. In response, the researchers introduced the photo elicitation method, a common qualitative design method where the researcher uses a photograph to elicit tacit and latent information (Harper, 2002). In this research, photo elicitation was intended to support a storytelling-based approach to describing and thinking about the importance of community. This included a series of publicly available photographs which were drawn by the university researchers from the Afri-Aus Care website, featuring familiar community events and activities, to maintain relevance to the community co-researchers and in response to their suggestions. The co-researchers were given time to look at the images and were asked questions that were included in the initial empathy map. Co-researchers focused on images that were most reflective of their engagement with community and with Afri-Aus Care.

**Figure 2.**
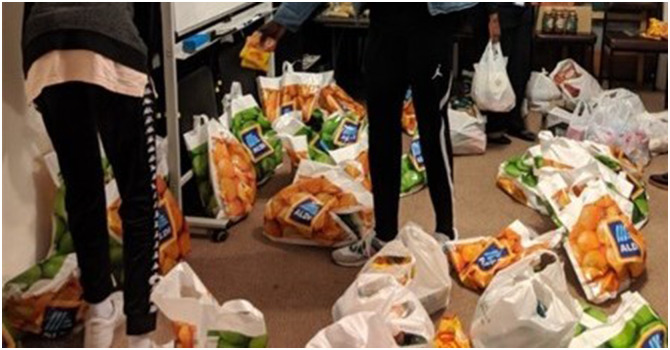
Image used in photo elicitation, showing community members packing food parcels for distribution during COVID-19 lockdown: © Afri-Aus Care, 2021.

For example, in one interview a co-researcher described their experiences of community, as a volunteer who had organized food parcels for community members during COVID-19 lockdowns (as shown in Figure 2).

As stated by a co-researcher:

So you’d come here, we are all from different backgrounds and different communities, it was amazing that we’d all somehow come together to help other people … It just felt, working together, there were sometimes when we would all laugh together, sometimes we would all be quiet working together. I felt, I think, a sense of belonging in what we were doing. I am trying to relate it to the whole program.

This highlights how the co-researcher drew on an image to tell their story of contributing to the community, while conveying the feelings they had of their community. The sense of togetherness, shared conversations, laughter, and silences, highlighted by the co-researcher, is associated with the important role that they were performing. They continue:

But we felt we were doing something for a good cause, we felt it was our responsibility to do that … I am feeding, not so much out of my pocket, but I am feeding a certain family. This could be my family, in another instance, you know? Yeah, I think the fact that we are doing it for a good cause felt good. And as you can tell [refers to image of bags lined up with food] everything is in order. And that was a good feeling as well. It’s not just someone is getting a bag and putting it on the ground, everyone’s like “I like what I am doing,” so it is being left in a good condition.

The co-researcher uses this part of the story to connect back to themes of UBUNTU, which they had described earlier in the interview. Through this, the image represents a practical demonstration of UBUNTU, and the narrative approach that they adopted helps to convey how the story of togetherness and community values was deeply underpinned by an understanding of UBUNTU.

Through shaping the co-design tools, to shift away from the text-based empathy map toward images, the focus group feedback ensured a more culturally relevant approach to data collection. Co-researchers were then able to use the images to tell stories of community, which they then related back to themes such as health and well-being, and articulate how UBUNTU informed their understanding and embodiment of community.

### Creating Together

Following Braun and Clarke’s (2012) approach to thematic analysis, we identified five themes in the recorded interviews related to the community’s priorities for the program that required further exploration. These ideas related to family and community involvement in the program, opportunities for youth leadership, embedding life skills training, incorporating gendered and religious sensitivities and inclusions, as well as the program’s relationship to the broader community. In a design process, it is generally at this point that a diverse range of stakeholders with varied expertise would come together in a workshop to create prototypes of suggested designs. However, the researchers recognized that we did not yet have confirmation from the community about our analysis, and further clarity and ideation about these priorities was required.

**Figure 3.**
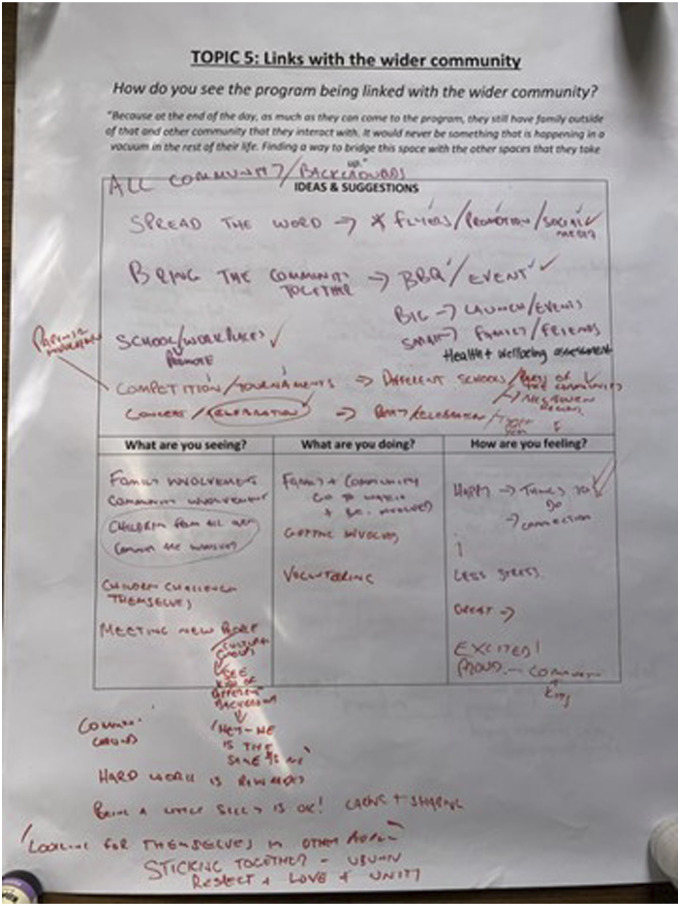
Co-researchers’ ideas for how the program could link with the wider community.

Community co-researchers came together in a final focus group to engage in a design charrette, working together in small groups to generate ideas or solutions to a specific question (Howard & Somerville, 2014). A design charrette includes a series of sequential, staged, and dynamic conversations, in which participants use craft and stationery materials to draw, write, map, or model ideas. In this research, the goal of the design charrette was for community members to clarify the priorities emergent in the thematic analysis and to generate ideas for how these priorities would be enacted within the program. Because of the co-researchers’ involvement in clarifying their priorities, the design charrette also performed an analytic purpose. We invited groups of two to three co-researchers to select a poster that was stuck to the wall at various spaces around the building, and after 5 min of generating ideas in response to one question, they moved to the next poster. Each poster contained a question, including “How could parents, family, Afri-Aus Care, schools, and other support services be involved in the program?” and “How do you see the program being connected with the wider community?” The posters also prompted community members to reflect upon what they would be seeing, doing, and feeling to generate ideas most aligned with their cultural values and practices of UBUNTU. Figure 3 is an example of community ideas for how the program could be linked with the wider community. Ideas that were shared or agreed by others were represented with ‘ticks’, such as ‘spreading the word’ through flyers, program promotion, and social media. Celebratory community-wide events that would bring the community together and include food were also a priority. The community-focused actions highlighted in Figure 3 reflect UBUNTU principles of moral responsibility (Mugambate & Chereni, 2020), care for others, harmony, a sense of obligation to participate (Abur & Mugumbate, 2022), belonging (Winschiers-Theophilus et al., 2012), and humanness in relationship to others (Metz, 2016).

## Discussion

Culturally humble CBCD was utilized to foreground community knowledge and expertise in design of the expanded Black Rhinos Basketball Program. To de-center Westernized, settler-colonial power operations as they exist within design discourses as well as the academy, we recognized that a humble approach required the establishment of processes for partnership, collaboration, and self-reflexivity. In the process, we learned that cultural humility requires ongoing and intentional attention to community and institutional power relations, a willingness to step away from traditionally defined researcher–community participant roles and methodological processes, and to nurturing curious, reciprocal, and authentic engagement with the community.

Cultural humility helped us to predict and recognize power operations at key points within the research process. Rather than positioning ourselves as ‘experts’, the examples above show that the co-researchers were engaged as partners to develop the conditions for the research as well as co-developing the methods which generated ideas for the program. Involving community members at various junctures enabled them to have a greater stake in decision-making, shifting the emphasis away from a researcher–community participant relationship (Goss et al., 2022) to co-authors and co-researchers (Luguetti et al., 2022). Aiming to understand issues of cultural importance, which were carefully integrated into the research design and engagement protocols through reflexive practice, helped us to challenge hierarchical power dynamics. While Yeager and Bauer-Wu (2013) suggest that reflexivity centers researchers rather than co-researchers, we considered this a necessary practice to locate how and where power would be dispersed with the community and how we would most effectively de-center ourselves and respond in emancipatory ways.

Reflecting on the relationships between the university researchers and co-researchers, we noticed that trust and rapport was easily established. This may have been due to the embodiment of UBUNTU within the community, the existing relationship between the university and the agency, and the ability for co-researchers to genuinely engage in both process and outcome. With their shared emphasis on mutuality and empowerment, the combination of CBCD, cultural humility, and UBUNTU established the conditions for an ongoing and reciprocal relationship between the researchers and community. The long-term character of these relational qualities offers an alternative perspective to traditional social research roles (Joubert & Webber, 2020) and the short-term nature of funded research projects, where the decision-making power is contained at an institutional level.

We also noticed that as university researchers, our commitment to authentically demonstrate trustworthiness and responsiveness within the community required us to be ‘unattached’ to a particular way of working. For example, the beginning stages of the research placed primacy on design tools and techniques rather than how the relationship with the co-researchers could inform the research. While co-design methodologies provide a framework for problem definition, idea generation, implementation, testing, and iteration of fit-for-purpose solutions (Winschiers-Theophilus et al., 2012) and rely on shared principles between designers and community stakeholders to guide processes, working toward community partnership required us to question our own assumptions and practices to maintain curiosity. This also extended to our data collection ideas, such as which of the community members would be involved in certain stages of the research process. Such flexibility can be challenging within a neoliberal, risk-averse academic context (Joubert & Webber, 2020), which values methodological expertise yet is incongruent with a culturally humble approach. Practicing methodological ‘curiosity’ was a significant step in both the design process and our relationship with the community, as it demonstrated a commitment to privilege community expertise, grounded in UBUNTU.

In co-designing the expanded Black Rhinos Basketball Program with the Afri-Aus Care community, we learned that a culturally humble approach has great potential for co-creating trusting, authentic, and collaborative partnerships in cross-cultural settings. There are, however, implications for its use in CBCD research. First, the use of Tascón and Gatwiri’s (2020) approach facilitated opportunities for self-reflexivity, which is arguably an absent stage within established design frameworks. Attending to our own impact on the design was essential for us to challenge potential actions that may bias design outcomes and, likewise, subvert power imbalances that facilitate Westernized ways of working. Second, Western researchers working with culturally diverse communities should include opportunities to establish connection and togetherness in the design process. Incorporating an intentionally unstructured component into community engagements deepens opportunities for authentic, mutual, and respectful relationships and can create conditions for partnership. Third and related to the second, it is essential to develop a trusted and reciprocal relationship with co-researchers. Tascón and Gatwiri (2020, p. 9) argue that cultural humility encourages an “ongoing, committed relationship” that focuses on care, respect, and mutual learning. Reflecting on our experiences from the interviews and focus groups, researchers should be prepared to “stop playing by the rule book” (Tascón & Gatwiri, 2020, p. 9), which in our case prompted greater openness of self through mutual exchange and embracing uncertainty in where the partnership may lead beyond the conclusion of the research.

## Limitations

Research that is funded and conducted within academic institutions inevitably must comply with key deliverables, timelines, and robust ethical processes. Research outcomes must therefore meet a range of competing demands beyond a focus on community wishes. While we aimed to embody principles of empowerment and partnership with the community, the nature of the academic research context suggests that power redistribution may only occur at a planning and operational level through contributions to the funding application and oversight of the design and day-to-day implementation of the program. At a process level, the university researchers presented proposed research methods and ideas for feedback and development, rather than the community having complete ownership over the research to begin with. Similarly, the university researchers made the decision to check the analysis with the co-researchers during the final workshop rather than involve them through co-analysis. We ultimately would have liked to include the co-researchers in the analysis of transcripts; however, due to ethical and resourcing constraints, including upholding the privacy of co-researchers (many of whom were known to each other), this was not ethically possible and would have required their participation without adequate compensation. In this sense, it seems the value of cultural humility is in our continued engagement with how we inherit and occupy power and how we aim to subvert institutional power for emancipatory purposes in explicit and experimental ways.

## Conclusion

The research we have undertaken with the Afri-Aus Care community has great potential in decolonizing CBCD practices, in that the combination of cultural humility, CBCD, and UBUNTU provides greater opportunities for community partnership, empowerment, and collaboration. In the case of our research, where the co-researchers are people with cultural knowledge and expertise, the UBUNTU philosophy provided a context where emphasis was placed on the relationship rather than the methodology. This relationality was well supported through our aim of cultural humility. As researchers, we still represented and enacted some power within the research process, yet we believe that a culturally humble approach can ideally be incorporated into co-design research where there are acknowledged differences in cultural identity within the design team. Ideally, design researchers and practitioners will recognize the potential for a culturally humble approach to create responsive, reciprocal, and authentic relationships with communities, challenge power imbalances, and move toward decolonizing their practice.
